# Effectiveness of management zones for recovering parrotfish species within the largest coastal marine protected area in Brazil

**DOI:** 10.1038/s41598-022-15990-1

**Published:** 2022-07-18

**Authors:** Pedro H. C. Pereira, Julia Caon Araujo, Gislaine V. Lima, Luís G. F. Côrtes, Erandy Gomes, Rafael A. Magris

**Affiliations:** 1 Projeto Conservação Recifal (PCR), Recife, Pernambuco Brazil; 2 Departamento de Geologia, Universidade Federal Do Rio de Janeiro, Avenida Athos da, Silveira Ramos, 274 - Cidade Universitária da Universidade Federal Do Rio de Janeiro, Rio de Janeiro, RJ 21941-916 Brazil; 3 Departamento de Oceanografia, Universidade Federal de Pernambuco, Recife, Pernambuco Brazil; 4 Chico Mendes Institute for Biodiversity Conservation, Ministry of Environment, Brasília, Brazil

**Keywords:** Conservation biology, Ecological modelling

## Abstract

The establishment of multiple zones offering different protection levels within a Marine Protected Area (MPA) can minimize social conflicts while maintaining associated biodiversity benefits such as fish population replenishment. Parrotfishes are among one of the most ecologically important reef fishes; yet extremely overexploited worldwide. In this context, well-designed priority management areas allowing no fishing activity (no-take zones) could help recover fish species, such as parrotfishes, through a MPA zoning process. Here, we tested this hypothesis by identifying the spatial configuration of zones that maximize the recovery of endangered parrotfish species (*Scarus trispinosus; Scarus zelindae*; *Sparisoma amplum*; *Sparisoma axillare*; *Sparisoma frondosum*) at the largest MPA in Brazil protecting nearshore coral reefs (MPA Costa dos Corais). We used parrotfish distribution data to produce species distribution models (SDMs) and combined them with conservation planning tools to delineate priority zones following a systematic approach. Then, we contrasted priority zones against non-systematic, newly designed no-take zones based on managers’ and stakeholders’ perspectives. After mapping the predicted abundance of each species within both zones based upon field surveys, we found that priority zones were more effective than non-systematic ones for the protection of two out of the five species: *Scarus trispinosus* and *Sparisoma amplum*. Thus, we considered that designing systematic zones was particularly relevant for increased protection of the two parrotfish species facing the largest decline. The prioritization analyses also showed that priority areas for parrotfish conservation following a systematic approach were mostly located surrounding and within no-take zones delineated by local stakeholders. The spatial overlap between systematic and non-systematic zones was of 38%. Hence, our study reinforces the importance of considering scientific information and methods (e.g., spatial distribution data and prioritization analyses) as a complementary strategy along with local stakeholders’ knowledge, for delineating and refining management zones within MPAs.

## Introduction

Coral reefs are one of the most ecologically relevant ecosystems on Earth. Yet, they are being subjected to multiple natural and anthropogenic impacts^1–3^. To mitigate the effects of human impacts such as overfishing, pollution, and global warming on coral reefs, the establishment of marine protected areas (MPAs) has been advocated as one of the most effective and widespread management strategies to date^4–6^. However, to increase species richness and biomass within their boundaries and benefit surrounding area through spillover and larval export, MPAs must be properly designed, implemented, and regulated^7–9^.

One of the critical steps in the MPA implementation process is the design of management zones^10,11^, which afford varying levels of restriction of human use within their boundaries. The establishment of multiple zones within an MPA can improve their overall effectiveness by minimizing social conflict between activities^12^, accommodate a myriad of conservation objectives in the planning process^5^, and optimize associated biodiversity and fishery benefits^11,13,14^. The science of conservation planning emphasizes the use of decision support tools to help identify the optimal spatial arrangement of zones^15^, which requires an understanding of the spatial distribution of habitats and species. Ideally, there will be also a large understanding of social and cultural context, and the integration of stakeholder’s perception into the zoning design process^14,16^. A robust design of management zones encompasses detailed information on biodiversity distribution and spatial representation of local uses, such as fishing and tourism, to identify areas where management actions are required while allowing human activities (i.e., multiple-use areas), and areas that are fully protected from all extractive and human uses (i.e., no-take areas) ^17–19^. Yet, such spatially explicit information on biodiversity and human uses is lacking for most of MPAs, especially in low and middle-income countries that are often facing a shortage of resources to support management activities.

Reef fishes are important biodiversity components that benefit from MPA success and are often considered model species for conservation and ecological studies^20–22^. Parrotfishes represent one of the most threatened fish groups worldwide with several overexploitation and local extinction recorded examples^23–25^.This group largely contributes to the dynamics of coral reefs, providing suitable settlement substrata for corals and mediating competition between corals and macroalgae^26^. Therefore, the lack of parrotfish grazing capacity could compromise MPAs efficiency and biodiversity recovery^27,28^. For instance, Mumby and Harborne^27^ suggested that marine reserves enhance the recovery of corals on Caribbean reefs and that parrotfishes play a large role in the recovery process of corals within MPAs. Nevertheless, little is known about parrotfish spatial distribution and about how the MPA zoning process could contribute to the recovery of parrotfish populations in the Southwestern Atlantic Ocean (SWA).

Management zones can be defined by managers’ and stakeholders’ perceptions within a participatory approach and without using decision-support tools (i.e., non-systematic design), or alternatively by a sequence of methodological steps that includes the usage of decision-support tools and the formulation of quantitative targets. In the latter approach, one of the most used systematic planning tools (i.e., Marxan) attempts to maintain or increase species population size by representing portions of their distribution within specific management zones^5,15^. Consequently, this approach can be derived from species distribution models (SDMs) in situations where distribution information is incomplete. SDMs are used to predict the distribution range of species based upon occurrence records and relevant environmental data^29^. Systematic and non-systematic approaches might thus be compared against each other using empirical data on the potential conservation benefits for relevant species, providing assistance in the MPA planning process. Direct measurements of species abundance could be used to evaluate the conservation benefits provided by management zones identified by systematic and non-systematic approaches in terms of helping promote biodiversity recovery over the implementation process of MPAs.

Here, we aimed to identify priority areas for conservation that would help recover threatened parrotfish species (*Scarus trispinosus; Scarus zelindae*; *Sparisoma amplum*; *Sparisoma axillare*; *Sparisoma frondosum*) at the largest coastal, multi-use MPA in Brazil (MPA Costa dos Corais), which allows biodiversity extractive uses such as fishing and tourism. To this end, we used a large field survey of parrotfish distribution and abundance with 94 sampling sites spatially distributed across the MPA and combined this information with modelling tools, such as SDMs and conservation planning software (i.e., Marxan) to identify priority areas for these species following a systematic approach. By doing so, we wanted to contrast the benefits of adopting this approach over the non-systematic design of zones in terms of their potential protection level provided against the decline of the parrotfish species due to overfishing. We evaluated these benefits by comparing the spatial overlap and the potential increase in the abundance of the parrotfish species offered by management zones defined by a systematic or a non-systematic approach. The non-systematic MPA zones were identified by managers and local stakeholders during the recent reviewing process of the MPA management plan (2017–2020). Ultimately, we aimed to provide insights into how effective the non-systematic zoning is in protecting these species and the required refinements. We posit that including empirical estimates of fish abundance into the MPA planning process will allow us to understand the benefits of each zone, and this inclusion could improve the overall MPA performance worldwide.

## Results

### Parrotfish density data

**Figure 1:**
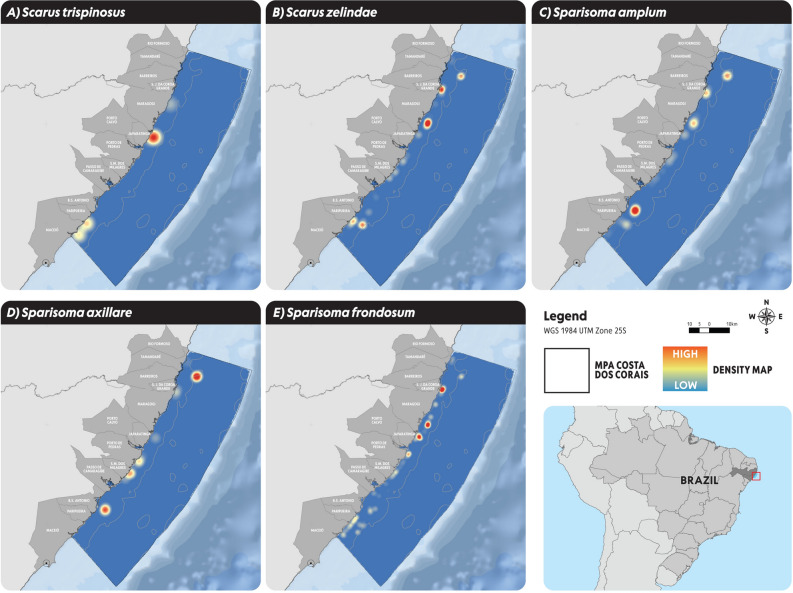
Parrotfish density data whitin MPA Costa dos Corais for imperiled parrotfish species: (**A**) *Scarus trispinosus*; (**B**) *Scarus zelindae*; (**C**) *Sparisoma amplum*; (**D**) *Sparisoma axillare*; (**E**) *Sparisoma frondosum*. Figure elaborated by the authors using Arcgis PRO.

For each parrotfish analyzed species, our field data presented higher density of *Scarus trispinosus* on the coast of Maragogi/Japaratinga and Paripueira/Maceió (Fig. 1A). For, *Scarus zelindae* a high density was observed at São José da Coroa Grande, South of Maragogi and Paripueira (Fig. 1B). A high density of *Sparisoma amplum* was recorded at the south of São José da Coroa Grande and Maragogi and at the coast of Barra de Santo Antônio (Fig. 1C) and a high density of *Sparisoma axillare* was observed at the north of São José da Coroa Grande, coast of Porto de Pedras/São Miguel dos Milagres and Barra de Santo Antônio (Fig. 1D). Finally, *Sparisoma frondosum* had a high density at the south of São José da Coroa Grande, Maragogi/Japaratinga and Porto de Pedras (Fig. 3E).

## Parrotfish species distribution modelling (SDM)

**Figure 2:**
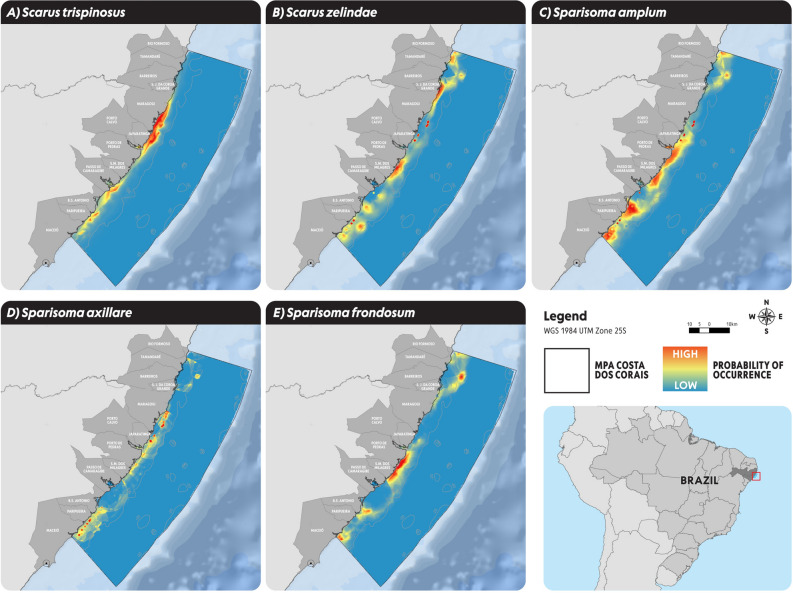
Parrotfish species distribution modelling (SDM) whitin MPA Costa dos Corais for imperiled parrotfish species: (**A**) *Scarus trispinosus*; (**B**) *Scarus zelindae*; (**C**): *Sparisoma amplum*; (**D**) *Sparisoma axillare*; (**E**) *Sparisoma frondosum*. Figure elaborated by the authors using Arcgis PRO.

Parrotfish species distribution modelling (SDM) demonstrated high probability of occurrence for *Scarus trispinosus* on the coast of Maragogi/Japaratinga, Passo de Camaragibe and Paripueira (Fig. 2A). For *Scarus zelindae* probability of occurrence was observed at Sao José da Coroa Grande and North of Maragogi, São Miguel dos Milagres/Porto de Pedras and Paripueira (Fig. 2B). A high probability of *Sparisoma amplum* occurrence was recorded at south of Japaratinga, Porto de Pedras/São Miguel dos Milagres and at the coast of Barra de Santo Antônio (Fig. 2C) and for *Sparisoma axillare* was observed at the south of Maragogi, Japaratinga, and south of Paripueira (Fig. 2D). Finally, *Sparisoma frondosum* had a high probability of occurrence at the south of Tamandaré/São José da Coroa Grande, Porto de Pedras/São Miguel dos Milagres and Maceió (Fig. 2E).

## Conservation prioritization

**Figure 3:**
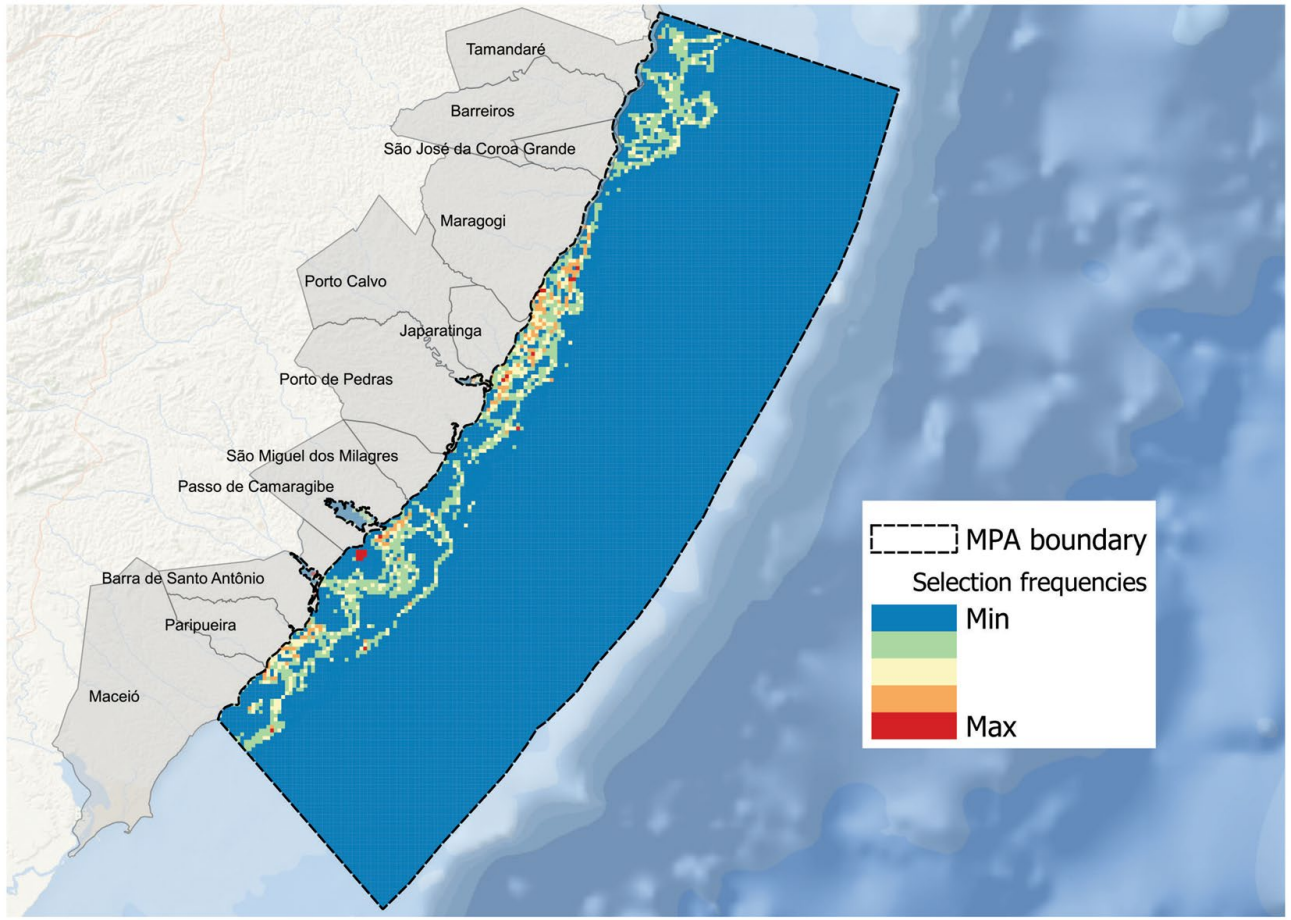
Selection frequency (0–100) output from Marxan, indicating more important areas (i.e., maximum selection frequency) for the conservation of the five parrotfish species. Figure elaborated by the authors using QGis 3.14.

The selection frequency output from Marxan indicates the importance of an area for achieving the conservation targets of all species. We found that mostly shallow areas closer to the coast were frequently selected across all runs (Fig. 3). The most frequently selected planning units were concentrated around a few, spatially restricted areas. That is an indication that there is no high flexibility achieving our conservation targets. Areas with high selection frequency were located within the estuary of the river Santo Antônio, near the estuary of the river Camaragibe, and on patchy areas between the central and northern parts of the MPA around the municipalities of Japaratinga and Maragogi.

## Evaluation of zoning effectiveness

**Figure 4:**
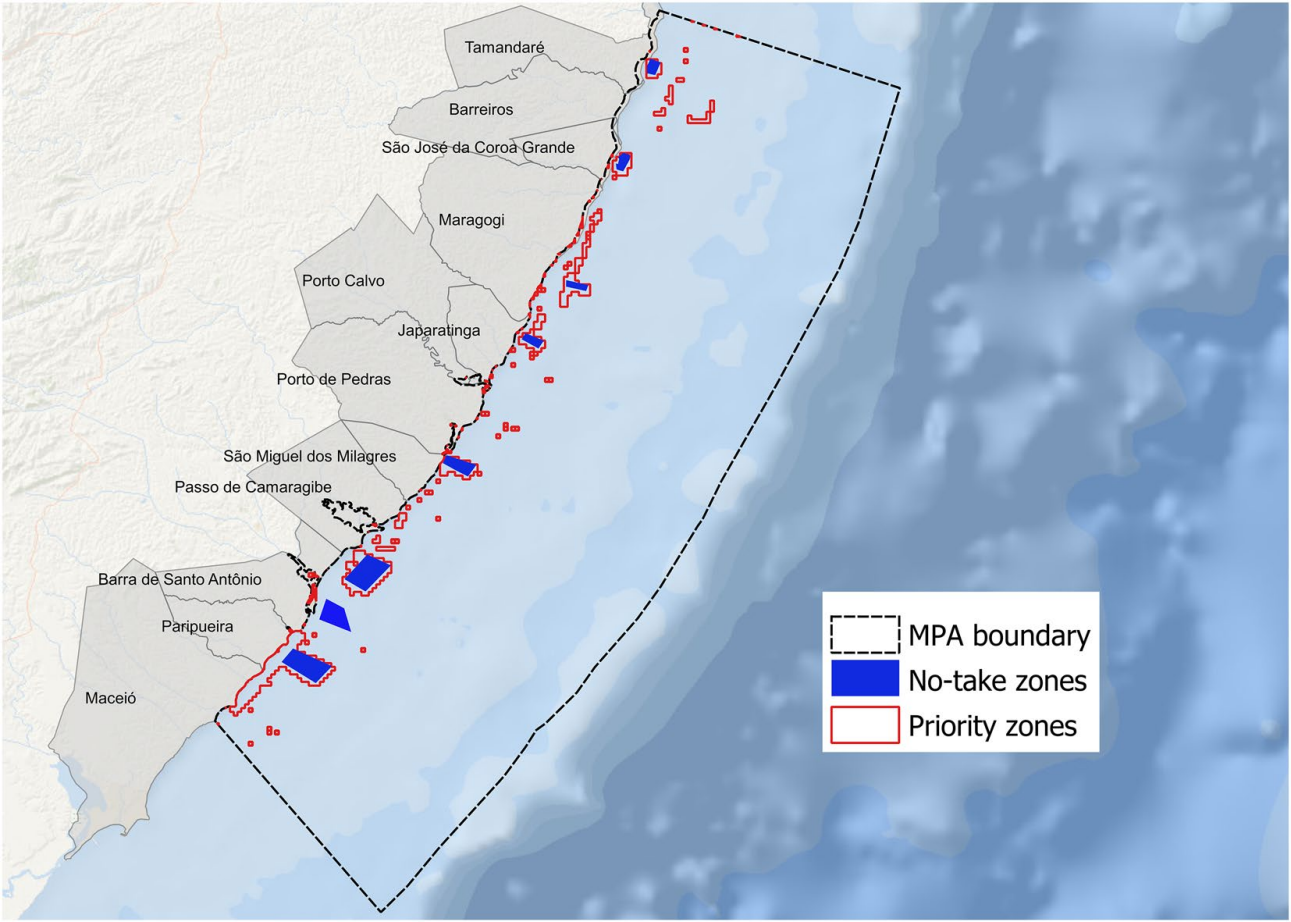
Priority zones identified using a systematic approach and existing no-take zones where fishing is currently not allowed within the MPA boundary. Figure elaborated by the authors using QGis 3.14.

Marxan’s best solution provides an indication of an individual solution that best achieved the conservation targets for parrotfishes at the MPA Costa dos Corais (i.e., “priority areas”). The priority areas were located mostly surrounding and within the existing no-take zones identified by stakeholders. The spatial overlap between priority zones and no-take ones was of 38%. Priority zones for parrotfish’s conservation summed up an area of about 504 km^2^ within the extent of the MPA Costa dos Corais (Fig. 4).

**Figure 5:**
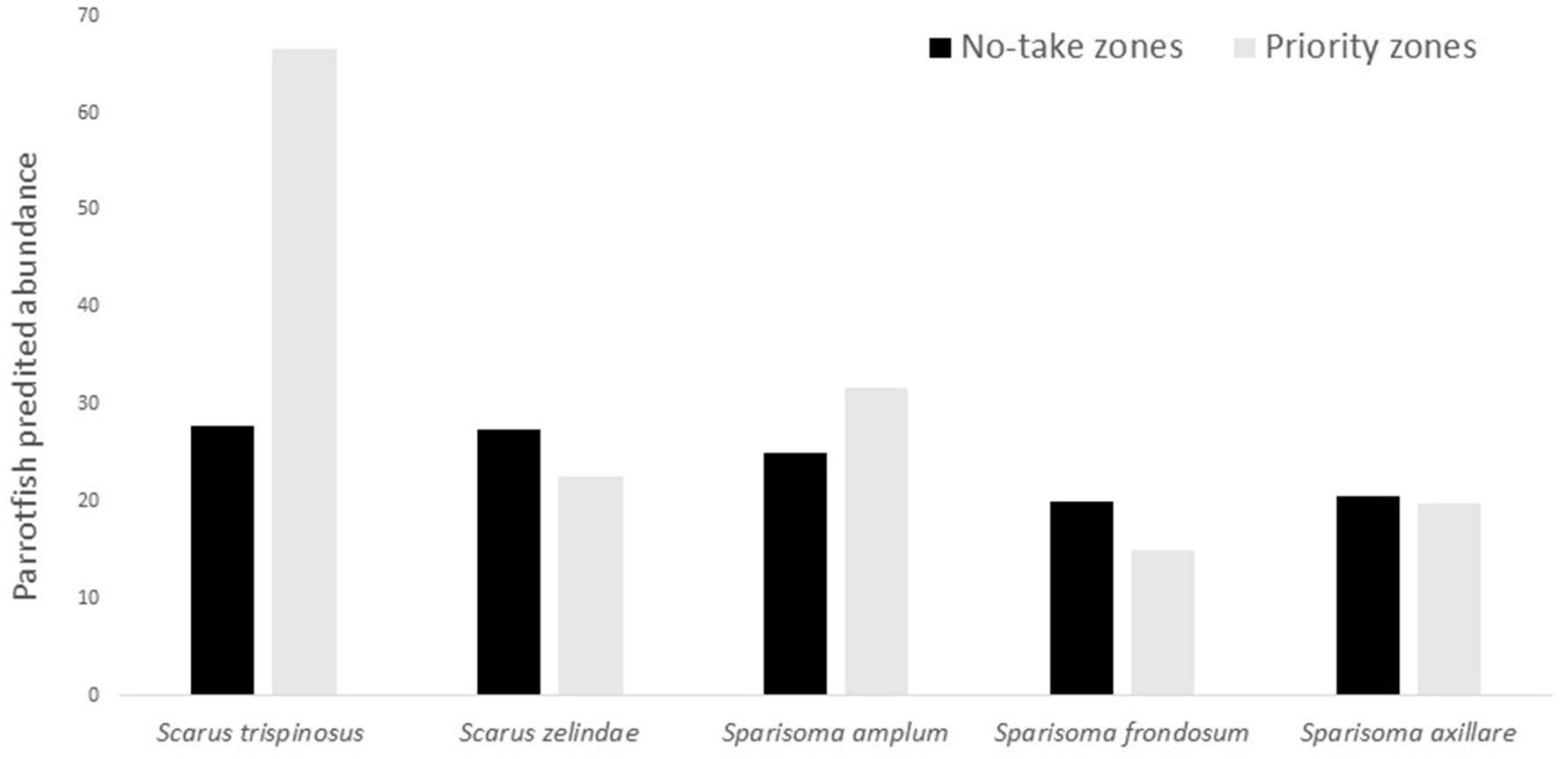
Predicted abundance for each parrotfish species within no-take and priority zones at MPA Costa dos Corais.

Based upon the predicted abundance of each species within zones, we found that priority zones were more effective than existing no-take zones identified by stakeholders for the protection of three species: *Scarus trispinosus*, *Sparisoma amplum* and *Sparisoma frondosum* (Fig. 5). Thus, the largest advantage of designing priority zones was to provide improved protection for these parrotfish species when compared to designed zones without a systematic approach.

## Discussion

Our study used spatial distribution and abundance data on parrotfish species to develop a prioritization approach for the largest Brazilian coastal MPA. Parrotfish fishing is an extremely important local community livelihood in this MPA^25,30^. Therefore, our study provides important baseline data for the long-term monitoring of these key species and supporting information for the refinement of management zones aiming to maintain parrotfish populations in the future and support local and national managers' conservation strategies. Based on Marxan conservation prioritization analyses, we identified that the priority areas for parrotfish conservation within the MPA were located mostly surrounding and within the no-take zones previously identified by stakeholders in a non-systematic approach^31^ and additional areas that were missed by the latter. The spatial overlap between priority zones and no-take zones was of 38% and the results showed that a both non-systematic and systematic approach are complementary strategies to design management zones entailing the risk of determining priority areas that are inappropriate for the recovery of species facing the largest declines in their abundance. Our findings support the value of stakeholder (fishers, local managers and researchers) knowledge and input into the more traditional “non-systematic” decision-making process. Additionally, our study highlighted the relevance of using distribution and abundance data of fish assemblages to refine no-take zones for the recovery of fish species and the optimal zoning strategies for coral reefs.

Conservation priorities derived from systematic and nonsystematic approaches can be complementary in terms of contributing to restoring depleted species such as parrotfishes as observed herein. For three species (*Scarus trispinosus*, *Sparisoma amplum* and *Sparisoma frondosum)* there was a large overlap between priorities derived from Marxan and developed based on local stakeholders. However, data-driven systematic approaches to conservation planning are necessary to pinpoint key areas that help recover species undergoing rapid decline, considered to be at high risk of local extinction^9,32^. Though the need to consider species abundance in the design of management zones has long been recognized^33,34^, only a few MPAs in Brazil have been consistently monitored with this type of biological data^35^, particularly for coral reef ecosystems. We found that this type of data was fundamental to provide guidance at identifying zones that are adequate to protect species in most need of conservation.

It was found that sitting the no-take zones implemented by stakeholders for sociopolitical reasons had some influence on the efficiency of the outputs in the study area. While 38% of the priority zones are currently fully protected, these zones are likely to generate community buy-in and strong support for conservation in the future. They can also set the stage for other parts of the MPAs to be fully protected in the future within an adaptive planning framework^36^. Given the importance of social acceptance in MPA success^37^, and the slow process of perceiving ecological and social benefits of protection^38^, the systematic approach based on ecological data presented here can be combined within a mixed approach as communities become more engaged in conservation and tourism activities over time. Furthermore, the location and extent of priority zones is likely to be heavily dependent on the choices of model parameters and criteria and data inputs, some of which could be somewhat biased.

Gathering ecological data requires setting up and running adequate monitoring programs which ensure that sampling strategy of target biodiversity components capture changes in biodiversity condition due to management actions^35^. Thus, long-term monitoring programs are required to monitor the effectiveness of zones and to distinguish the status and changes of coral reef assemblages due to disturbances and management strategies^39,40^. Although we assembled monitoring data for a few species that could help us gain an understanding of the ecological benefits of adopting systematic and non-systematic approaches, the similarity of ecological characteristics between species means that it would have been wise to consider ecological data of other fish and benthic species in the future. Owing to the widespread shortfalls in funding worldwide^41,42^, conservation managers must decide where the investment is needed, whether in the establishment of new conservation zones or the enforcement of the existing ones. In this sense, we suggest that although monitoring data is desirable in conservation planning, spatial adjustments regarding the configuration of zones would provide progressively improvements of parrotfish species conservation.

The is as urgent call to understand the status of Brazilian reef fish populations and design appropriate management and conservation strategies^9,43,44^. Many species of herbivorous (Scarinae family) are currently overexploited and threatened with extinction worldwide^23,25,44^. Using traditional stock assessment models, Frédou^45^ also found that the most common Lutjanidae (5 species) in northeastern of Brazil were fully or overexploited in fished areas. The absence of large carnivores increases the vulnerability of herbivorous fish to overfishing in coastal communities^46^. Recent studies demonstrated the reduction of herbivorous fish populations, mainly Scaridae, due to overfishing^26,47–51^. For instance, based on declines measured at various locations, the global population of *Scarus trispinosus*, a species analyzed in the present study,is estimated to have decreased by at least 50% in the last 20 to 30 years (three generations in duration) ^25,52,53^. Conversely, urgent action including priority conservation modelling for MPA zoning such as presented herein and fisheries management plan are necessary to protect parrotfishes.

A new management strategy in force since June 2019 in Brazil, has been introduced to deter the overfishing of parrotfish species^54^. This innovative strategy, “*inverted management*”, allows the capture of endangered species inside management areas, such as partially-protected marine areas (MPAs), while it remains banned elsewhere. However, the adequate implementation of this strategy depends on the adoption of a series of challenging management measures such as co-management, surveillance, high level fishery statistics data and long-term monitoring. Currently, MPA Costa dos Corais is working on the fishing regulation for three parrotfish species, *Scarus trispinosus*; *Sparisoma amplum*, *Sparisoma frondosum*. The management plan will include size catch restrictions, quota per fish, and catch ban on specific periods (e.g., reproduction seasons). For instance, for the endangered and endemic parrotfish *S. trispinosus* as suggested by Pereira^25^ catch would be only allowed during the day and for individuals larger than 40 cm and weighting over 3 kg. A season ban would be also recommended during February–March and August–September (spawning peaks). Lastly, fish may be landed eviscerated but never in fillets which makes identification of the species difficult during monitoring.

It is worth mentioning that several socioeconomic aspects such as touristic pressure, fishing activities, and pollution, including sewage discharge, must be considered during the planning and evaluation of management and monitoring actions of MPAs. According to MPA Costa dos Corais Public Use Plan^31^, the MPA received around 315,000 visitors in 2019, 85% only in the natural pools of Maragogi. The numbers refer only to visits in areas with visitation control, i.e., the natural pools of Maragogi, Japaratinga and Paripueira and the visits to manatees in Porto de Pedras. Yet, this number is certainly underestimated and, according to the management of the MPA the number of visitors may be in the order of millions per year^31^ and must be considered during zoning strategies. Additional local factors that should also be considered include: proximity to sugarcane plantations, which increase the risk of pollution^55,56^; contamination by oil and oil products^57^; invasive species such as sun coral and lionfish^58–60^. Hence, all this additional socioeconomic and ecological impacts must be properly addressed for effective MPA zoning process and associated species conservation.

## Methods

### Study area

**Figure 6:**
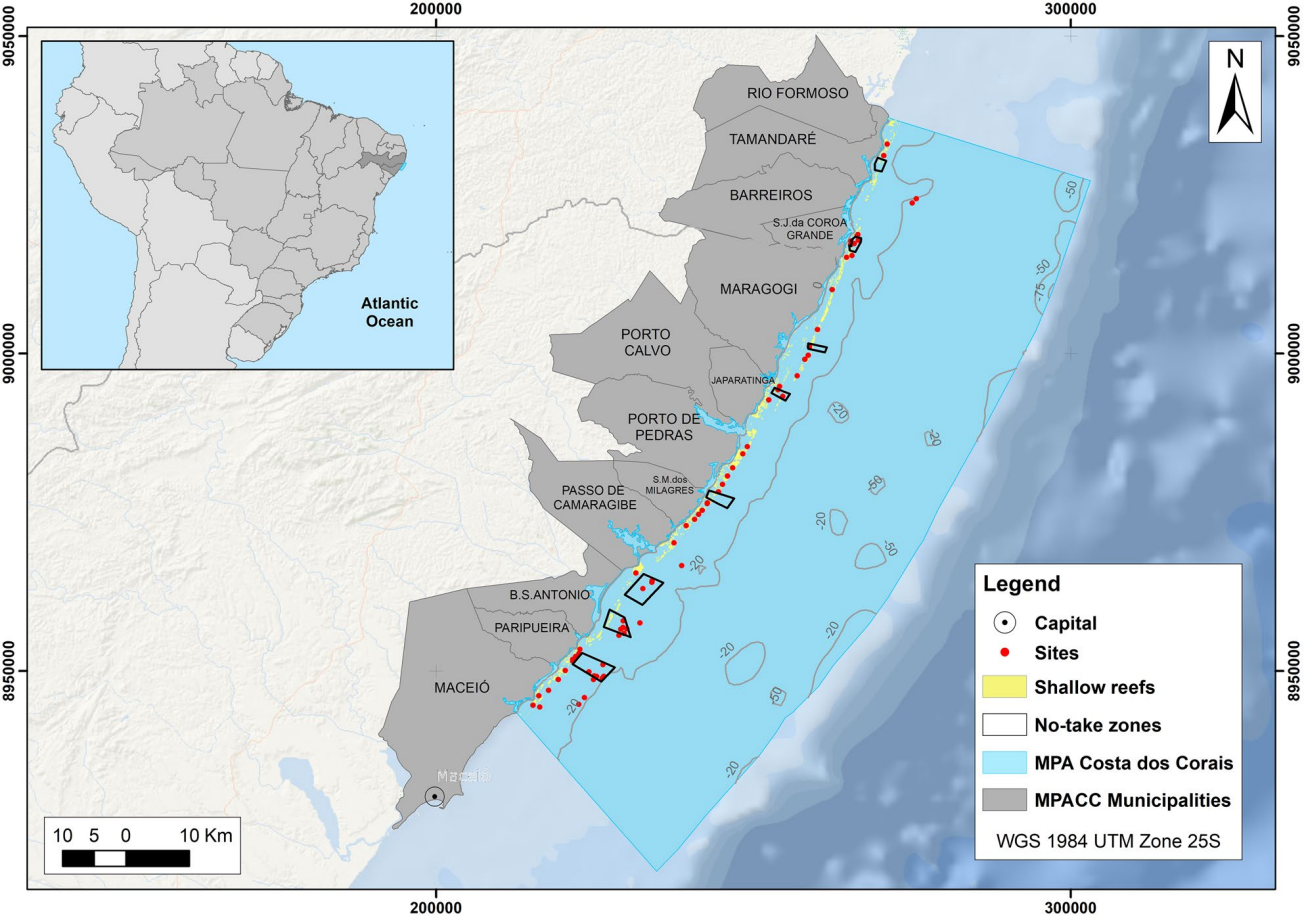
– Map of MPA Costa dos Corais highlighting the 94 sampling sites where field data for parrotfish species was collected. Figure elaborated by the authors using Arcgis PRO.

The present study was performed at MPA Costa dos Corais, the largest coastal multiple-se marine protected area in Brazil, created in 1997 to protect coral reef ecosystems on Brazilian waters. This MPA stretches from 120 km in northeastern Brazil encompassing two states and 12 municipalities (Fig. 6). MPA Costa dos Corais covers a large range of different ecosystems such as shallow reefs, mangroves, seagrass beds, rhodolith/sponge beds and mesophotic reefs from the coast to the break of the continental shelf^25,61,62^.

The multiple use MPA corresponds to an IUCN category VI protected area, where sustainable use is admitted according to its management plan, published in 2013^63^ and recently revised^31^. This new version of the management plan was elaborated by managers in partnership with local NGOs, researchers, fishers, and touristic trade, and became official recently^31^. The zoning plan was considered the best strategy to promote coral reefs conservation locally with some areas indicated as “no-take zones” (where all human activities, except research, are prohibited), touristic areas (where only low impact visiting activities are allowed), and large multiple-use zones (where fishing and tourism activities are allowed).

### Parrotfish field data

Underwater observation was carried from 2017 to 2020 in 94 sites distributed along the MPA Costa dos Corais to determine the spatial distribution and abundance of five parrotfish species (*Scarus zelindae*; *Scarus trispinosus*; *Sparisoma amplum*; *Sparisoma axillare*; *Sparisoma frondosum*) (Fig. 6). Field data was collected in belt transects of 20 m long by 5 × 5 m wide (2.5 m on the right and 2.5 m on the left), with a minimum of 4 linear transects performed per site^64^.

## Parrotfish species distribution modelling (SDM)

The methodology for creating the maximum entropy models followed the parameters established by Dalapicolla^65^. Maximum entropy models for habitat prediction use georeferenced occurrences and environmental variables such as mean temperature, mean precipitation, calcite content, salinity, etc^66,67^. The objective was to predict which areas within the region meet the requirements of the species' ecological niche and, therefore, are part of the species' potential distribution^68^. The potential distribution describes suitable conditions for species survival and therefore of greater conservation importance^69–71^. Several studies have used maximum entropy (Maxent) methodology to predict global and local habitats^72–77^.

To generate the model, data files were converted to Maxent software formats. Hit points were converted from shapefile to .csv and environmental variables from .tiff to .asc. The environmental variables files (bathymetry, transparency, coral cover and algae cover) (Table S1) were entered with the same cell/pixel size, rows and columns. The geoprocessing steps were developed in Arcgis 10.8 software.

SCUBA diving was used to survey a 20 m × 5 m belt transect (2.5 m on the right and 2.5 m on the left) for coral cover and algae cover on the same analyzed parrotfish sites (Fig. 6). Point Intercept Transect (PIT) was used where data was recorded along the central transect line ((Hill and Wilkinson, 2004; Leão et al., 2016) (see Pereira et al., 2022 for methodological details). Coral cover and algae cover was calculated as a percentage of cover for each belt transect.

In the current model, all environmental variables were continuous, and pixels represented values (e.g., bathymetry). The options CREATE RESPONSE CURVES and DO JACKKNIFE TO MEASURE VARIABLE IMPORTANCE were selected to generated a graph for each variable indicating the behavior of the probability of occurrence of the species with the increase in the value of the variables and the calculation of the importance of each variable for the model. For the species *Scarus zelindae* and *Sparisoma axillare* with a few samples above 80, the AUTO FEATURES option was selected. From 15 to 79 occurrences (*Sparisoma amplum* and *Sparisoma frondosum*) linear, quadratic and hinge features were selected. Finally, for species below 10 samples (*Scarus trispinosus*) the linear option was applied.

For each species, 10 models (replicates) were created, which were later analyzed according to statistical parameters in search of the best indexes to choose the final model. Processing returns a .csv table with the parameters of each model. The mean values of the following parameters are analyzed: AUC Test; Minimum training presence logistic threshold; Minimum training presence test omission; Minimum training presence binomial probability; 10 percentile training presence logistic threshold. Then, in the Arcgis software, the models were cut by the threshold values, opting for the 10% threshold value.

The quality of the models was statistically verified from the parameters determined in the bibliography for evaluation (see Supplementary Material), mainly the AUC test. The AUC test is a threshold independent measure of model performance compared to the null hypothesis for prediction (Fielding and Bell 1997). Models with AUC ≤ 0.50 are considered to perform poorly, possibly worse than a random prediction, while higher AUC values, preferably above 0.7, indicate better prediction results. The SDM models for parrotfish species showed AUC values between 0.92 and 0.97.

## Spatial prioritization analyses

We performed a conservation prioritization to reflect the potential benefit of priority areas in the recovery of parrotfish species. We used the software package prioritzr (Marxan function) to identify spatial priorities that meet percentage target for each species, while accounting for the different level of protection required to each species, and minimizing the total cost of the selected areas, with area as a measure of cost. Marxan uses simulated annealing to solve the minimum-set problem^78^. Because we wanted to compare the spatial configuration of different type of zones, and their potential benefit to recover fish populations, we did not allow non-systematic zones to be necessarily selected by Marxan (i.e., they were not locked in the analysis; that is also because the non-systematic zones had not been formally implemented at the time of the analysis). We used 1 km square cells as our planning units (N = 17,099), which are the minimum area that could be potentially selected for protection. The planning-unit size reflected the scale of the species distribution data.

We defined species-specific targets based on natural rarity ($$NR_{i} )$$, vulnerability to threats $$\left( {VL_{i} } \right)$$, and past range reduction through habitat loss ($$RP_{i} )$$. Natural rarity was perceived as the distribution size of each species within the MPA as defined by the binary results of the SDMs. To estimate vulnerability, we extracted the scores of cumulative threats facing each species under consideration within their distribution area inside the MPA as described in Magris^9^. The threats considered in the analyses included fishing, land-derived pollution, ocean-based pollution, climate change, and coastal development and their scales were previously normalized to a range 0–1 within the MPA boundaries to allow comparison. We calculated the mean cumulative threat score for all planning units within their present distribution. To estimate the habitat loss, we summed up the number of pixels from the binary distribution modelling results in which the species had null abundance according to the survey field data (i.e., where the species is known to be absent). Each factor individually was normalized to the reference range zero to one (where one represents the species with the smallest distribution, facing the highest level of threat, and associated with the largest potential reduction in population size; and zero represents the species with the largest distribution, facing the lowest level of threat, and found to have the smallest potential reduction in population size), as follows:

$$NR_{i} = \left( {SDM_{max} - SDM_{i} } \right) \div SDM_{max}$$

where $SDM_{max}$ is the largest SDM among all species, and $SDM_{i}$ is the distribution size for the species under consideration.

$$VL_{i} = 1 - \left[ {\left( {CT_{max} - CT_{i} } \right) \div CT_{max} } \right]$$

where $CT_{max}$ is the highest cumulative impact score among all species, and $CT_{i}$ is the cumulative impact score for the species under consideration.

$$RP_{i} = 1 - \left[ {\left( {RP_{max} - RP_{i} } \right) \div RP_{max} } \right]$$

where $RP_{max}$ is the largest reduction in the range among all species and $RP_{i}$ is the past reduction in the range for the species under consideration.

We calculated the target for each species as the sum of all factors, ensuring that all species had a minimum target of 10% and a maximum of 30% (Table 1). We followed standard practices to calibrate the boundary length modifier (BLM) and set it to 1. We considered “priority zone” those planning units selected by Marxan’s best solution.

**Table 1:** Targets used in the prioritization analyses for four parrotfish species: *Scarus trispinosus; Scarus zelindae*; *Sparisoma amplum*; *Sparisoma axillare*; *Sparisoma frondosum*).

Species	Target (proportion of the distribution area)
*Sparisoma amplum*	0.20
*Sparisoma axillare*	0.10
*Sparisoma frondosum*	0.20
*Scarus trispinosus*	0.30
*Scarus zelindae*	0.20

## Evaluation the conservation effectiveness of MPA zones

After identifying priority zones by the systematic approach, we examined the spatial overlap between this zone, as defined above, and the no-take zones defined by a non-systematic approach.

Lastly, we estimated the potential benefit to fish populations of each species provided by each type of zone. Because we lacked abundance data for the whole zone areas and due to the spatial autocorrelation observed in our sampling sites, kriging could be used as a method to interpolate density data and predict density levels of each species in unsampled areas of the MPA. By doing so, we created an approximation of a predictive map of abundance of each species and for all planning units belonging to each zone. . Prior to interpolation, density data were normalized with the necessary resolution for better visualization. Interpolation procedures were generated using the Kernel Density tool^79^ in raster format on Arcgis 10.8 for each parrotfish species: *Scarus trispinosus; Scarus zelindae*; *Sparisoma amplum*; *Sparisoma axillare*; *Sparisoma frondosum.* Ideally, it would be more robust to create formal predictive maps of density through more sophisticated analysis, but gathering more detailed information on environmental predictors of abundance was beyond the scope of this study and should be incorporated into future modeling attempts.

**Figure 7:**
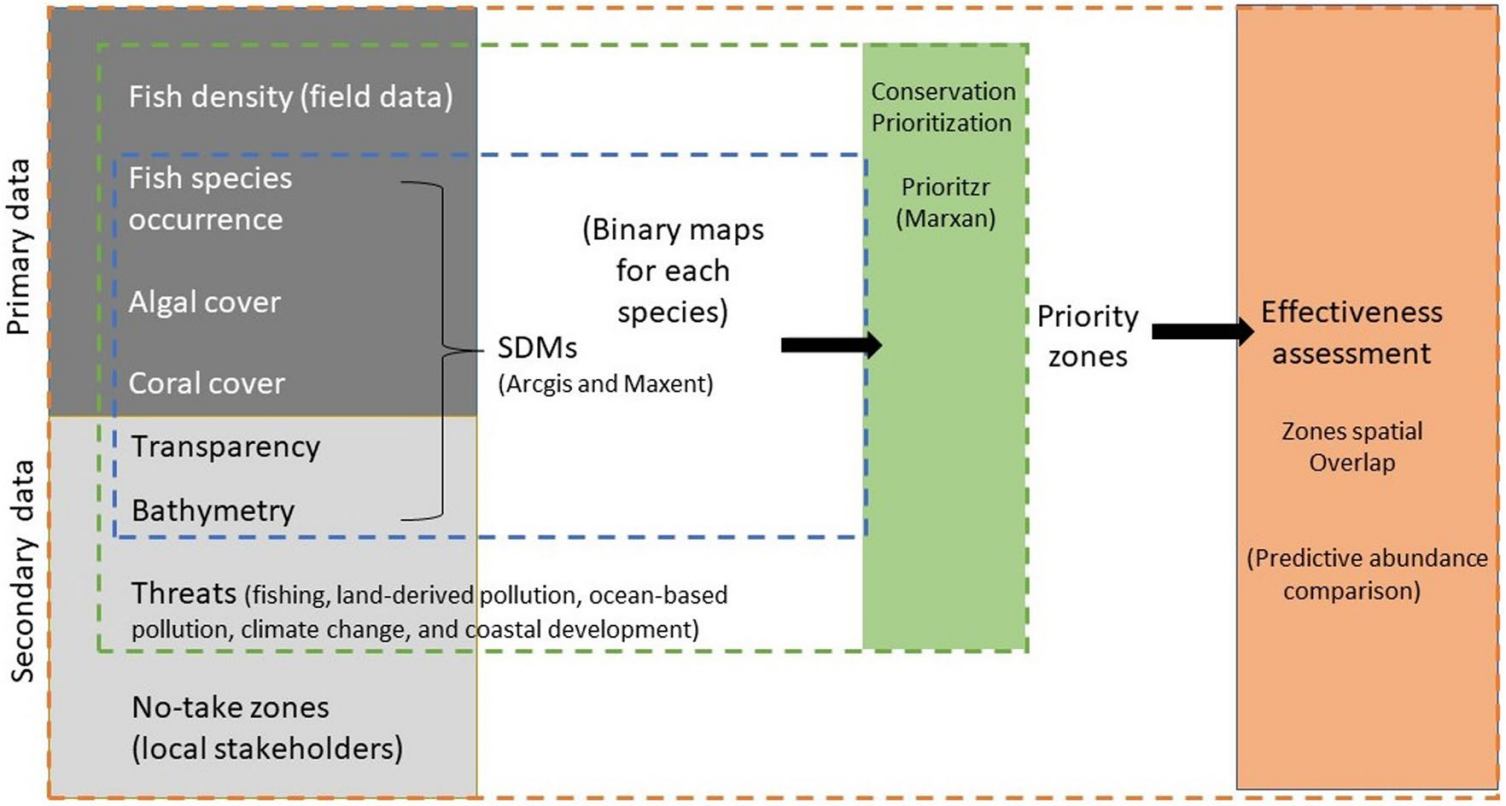
Flowchart of the methodological steps and types of data involved in the analysis of assessing the effectiveness of systematic and non-systematic zones.

A flowchart was elaborated to help readers understand the different pieces of the presented analysis and how they fit together to accomplish the present study aims (Fig. 7).

## Supplementary Information

Supplementary Information 1.

Supplementary Information 2.

Supplementary Information 3.

Supplementary Information 4.

Supplementary Information 5.

Supplementary Information 6.

Supplementary Information 7.

Supplementary Information 8.

## Data Availability

Raw data from this manuscript has been included in supplementary files section.
