# Supplementary material for: Effectiveness of management zones for recovering parrotfish species within the largest coastal marine protected area in Brazil
Source: Sci Rep. 2022 Jul 18;12:12232. doi: 10.1038/s41598-022-15990-1 (PMC9293920; doi:10.1038/s41598-022-15990-1)
Supplement: Supplementary file 7 — Supplementary Information 7. [file 41598_2022_15990_MOESM7_ESM.docx]

Effectiveness of management zones for recovering parrotfish species within the largest coastal marine protected area in Brazil

– ODMAP Protocol –

Pedro Henrique Cipresso Pereira, Julia Caon Araujo, Gislaine Vanessa Lima, Luís Guilherme França Côrtes Silva, Erandy Gomes Silva, Rafael Almeida Magris

2022-06-02

## Overview

#### Authorship

Contact : [pedrohcp2@yahoo.com.br](mailto:pedrohcp2@yahoo.com.br)

Study link: <https://www.speciesconservation.org/case-studies-projects/greenback-parrotfish/8526>

#### Model objective

Model objective: Mapping and interpolation

Target output: suitable vs. unsuitable habitat

#### Focal Taxon

Focal Taxon: parrotfish

#### Location

Location: Marine Protected Area Costa dos Corais, Northeast of Brazil

#### Scale of Analysis

Spatial extent: -35.60530, -35.07295, -9.56491, -8.70052 (xmin, xmax, ymin, ymax)

Spatial resolution: 0,1

Temporal extent: single time slice

Temporal resolution: 2017-2020

Boundary: political

#### Biodiversity data

Observation type: field survey

Response data type: point occurrence, presence/absence

#### Predictors

Predictor types: topographic, habitat

#### Hypotheses

Hypotheses: Parrotfish fish occupy regions preferentially with corals and with availability of algae Well-designed priority conservation areas allowing no fishing activity (no-take zones) could help recover fish species, such as parrotfishes, species through a MPA zoning process

#### Assumptions

Model assumptions: We used parrotfish distribution data to produce species distribution models (SDMs) and combined them with conservation planning tools to delineate priority zones following a systematic approach Species are at (pseudo-) equilibrium with their environment Species show largely similar responses to the transect. Sampling is adequate and representative (and any biases are accounted for/corrected)

#### Algorithms

Modelling techniques: maxent

Model complexity: We fitted MaxEnt and single-visit site-occupancy models to the field data. MaxEnt was chosen due to competitive performance on small sample sizes and ease of use. Easy association of Maxent with other software like Arcgis 10.8

Model averaging: MaxEnt models were built with automatic Features (Scarus Zelindae, Sparisoma axillare ); Linear, Quadratic e Hinge features (Sparisoma amplum e Sparisoma frondosum and linear to Scarus trispinosus. We combine the models for all species to form ensemble predictions

#### Workflow

Model workflow: A. Background data: We chose the environmental variables (background) as indicated by Merow et al. 2013. based on the spatial scale and environmental conditions that could establish the presence of parrotfish. B. Features: Non-categorical variables were chosen. We include all predictors in the model letting the algorithm decide which ones are important. In machine learning methods, high collinearity is less of a problem in studies with the objective of predictive accuracy of attendance (Merow et al. 2013), which is our case. C. Regularization: We decrease the model complexity by selecting the one FEATURE CLASS (FC) and a specific REGULARIZATION MULTIPLIER (RM) value taking into account the number of occurrence points and environmental variable, according to Merow et al. 2013. For species (Scarus Zelindae, Sparisoma axillare and model for all species together) with a number of samples above 80, the AUTO FEATURES option was selected. From 15 to 79 occurrences (Sparisoma amplum and Sparisoma frondosum) the Linear, Quadratic and Hinge features options were selected. Finally, for species below 10 samples, the linear option (Scarus trispinosus). The specific settings for the dataset above 14 samples in replicated run type was Bootstrap (Scarus Zelindae, Sparisoma axillare, Sparisoma amplum, Sparisoma frondosum and model for all species together). For the data set smaller than 14 points, the configurations of replicated run type was Crossvalidate (Scarus trispinosus). D. Samples bias: we limit our model to the area of ​​environmental protection as it is our area of ​​interest. MaxEnt randomly selects background (pseudo-absence) points within the study area. However, we do not use Bias Grid to indicate areas within our study region so that they are more sampled when MaxEnt selects these points. This avoids biased sampling. E. Types of output: Here we opt for the type of logistic output to determine the probability of presence to increase the quality of the calibration. F. Evaluating models: We evaluate models by their AUC value to determine how the model distinguishes between presence and absence. We check the column values ​​of each model in the row (average) for Test AUC, Minimum training presence logistic threshold, Minimum training presence test omission, Minimum training presence binomial probability, 10 percentile training presence logistic threshold. Then, in Arcgis software, the models were cut by the threshold values, choosing the threshold value of 10%, the most used value in the literature. In Arcgis, the following steps were performed: (1) Conversion of the .asc file to tiff: Conversion Tools > To Raster >ASCII to raster; (2) Creation of the binary model (presence/absence) in the Raster Calculator: average_model >= threshold value of 10%; (3) Creation of the final model in the Raster Calculator: 'binary_model' * 'initial_raster'. The binary map has inadequate pixels for the presence of the species with a value of 0 and with adequate pixels with values ​​of 1. Then we multiply the binary map by the original raster and generate the final map.

#### Software

Software: Maxent v3.3.3 (<http://www.cs.princeton.edu/~schapire/maxent/>) and Arcgis 10.8

<Code availability>

Data availability: The raw data are available as a supplementary file accompanying the article.

## Data

#### Biodiversity data

Taxon names: Scarus trispinosus; Scarus zelindae; Sparisoma amplum; Sparisoma axillare; Sparisoma frondosum

<Taxonomic reference system>

Ecological level: species, populations

Data sources: Survey data collected in the field between 2017-2020.

Sampling design: We have been using 20 m belt transects for MPA fish community monitoring in the last years and it is assumed as a standardized methodology for fish community in Brazilian waters (Pereira et al., 2018; Roos et al., 2019; Cordeiro et at al., 2021

Sample size: (94 sites)

Clipping: Pernambuco/Alagoas, Brazil

Scaling: Occurrence points were converted from shapefile to .csv and environmental variables from .tiff to .asc. The table of occurrence points was inserted according to the example format: “zel,242898.4215,8976445.763”, that is, “species,x coordinate, y coordinate” format in the same column. The environmental variables files were entered with the same cell/pixel size, rows and columns. For this, the bathymetry file was resampled to cell size (99,37 X 99,37).

Cleaning: For the maxent we used the positive values ​​of abundance for each species as the number of occurrences. For example, values ​​greater than zero at each site were counted as occurrence points. Zero abundance values ​​were not considered as occurrence points.

<Absence data>

<Background data>

#### Data partitioning

Training data: No data partitioning was applied; model performance was assessed using bootstrapresampling (n = 10 replicates)

Validation data: The average values ​​of the following parameters are analyzed: - AUC test - Minimum training presence logistic threshold - Minimum training presence test omission - Minimum training presence binomial probability - 10 percentile training presence logistic threshold Then, in Arcgis software, the models were cut by the threshold values, choosing the threshold value of 10%, the most used value in the literature. In Arcgis, the following steps were performed: (1) Conversion from .asc file to tiff: Conversion Tools > To Raster >ASCII to raster (2) Creation of the binary model (presence/absence) in the Raster Calculator: average_model >= threshold value of 10% . (3) Creation of the final model in the Raster Calculator: 'binary_model' * 'initial_raster'

#### Predictor variables

Predictor variables: Topography: : Depth Habitat: water clarity - transparency, coral cover and algae cover

Data sources: ICMBIO, Brasil.

Spatial extent: -35.60530, -35.07295, -9.564919, -8.704963 (xmin, xmax, ymin, ymax)

Spatial resolution: 99,37m

Coordinate reference system: WGS 1984 UTM ZONE 25S

<Temporal extent>

#### Transfer data

<Data sources>

<Spatial extent>

<Spatial resolution>

<Temporal extent>

<Models and scenarios>

<Quantification of Novelty>

## Model

#### Multicollinearity

<Multicollinearity>

#### Model settings

<maxent>

<Model settings (extrapolation)>

#### Model estimates

<Coefficients>

#### Model selection - model averaging - ensembles

Model averaging: We took an information-theoretic approach to model averaging for each model class, considering all combinations of covariates.

Model ensembles: Within model classes, candidate models were ranked based on their AUCc scores, Minimum training presence logistic threshold, Minimum training presence test omission, Minimum training presence binomial probability; 10 percentile training presence logistic threshold

#### Analysis and Correction of non-independence

<Spatial autocorrelation>

#### Threshold selection

Threshold selection: The average values ​​of the following parameters are analyzed: - AUC test - Minimum training presence logistic threshold - Minimum training presence test omission - Minimum training presence binomial probability - 10 percentile training presence logistic threshold Then, in Arcgis software, the models were cut by the threshold values, choosing the threshold value of 10%, the most used value in the literature. In Arcgis, the following steps were performed: (1) Conversion from .asc file to tiff: Conversion Tools > To Raster >ASCII to raster (2) Creation of the binary model (presence/absence) in the Raster Calculator: average_model >= threshold value of 10%. (3) Creation of the final model in the Raster Calculator: 'binary_model' * 'initial_raster'

## Assessment

#### Performance statistics

Performance on training data: AUC, TSS

Performance on validation data: AUC

Performance on test data: AUC

#### Plausibility check

<Response shapes>

<Expert judgement>

## Prediction

#### Prediction output

Prediction unit: Predictions of relative probability of presence expressed on a continuous scale.

Post-processing: In Arcgis software, the models were cut by the threshold values, choosing the threshold value of 10%, the most used value in the literature. In Arcgis, the following steps were performed: (1) Conversion from .asc file to tiff: Conversion Tools > To Raster >ASCII to raster (2) Creation of the binary model (presence/absence) in the Raster Calculator: average_model >= threshold value of 10%. (3) Creation of the final model in the Raster Calculator: 'binary_model' * 'initial_raster'

#### Uncertainty quantification

<Scenario uncertainty>

<Novel environments>
