# Supplementary material for: Effectiveness of management zones for recovering parrotfish species within the largest coastal marine protected area in Brazil
Source: Sci Rep. 2022 Jul 18;12:12232. doi: 10.1038/s41598-022-15990-1 (PMC9293920; doi:10.1038/s41598-022-15990-1)
Supplement: Supplementary file 8 — Supplementary Information 8. [file 41598_2022_15990_MOESM8_ESM.docx]

**MPA Costa dos Corais layers**

**Pereira et al., 2022**


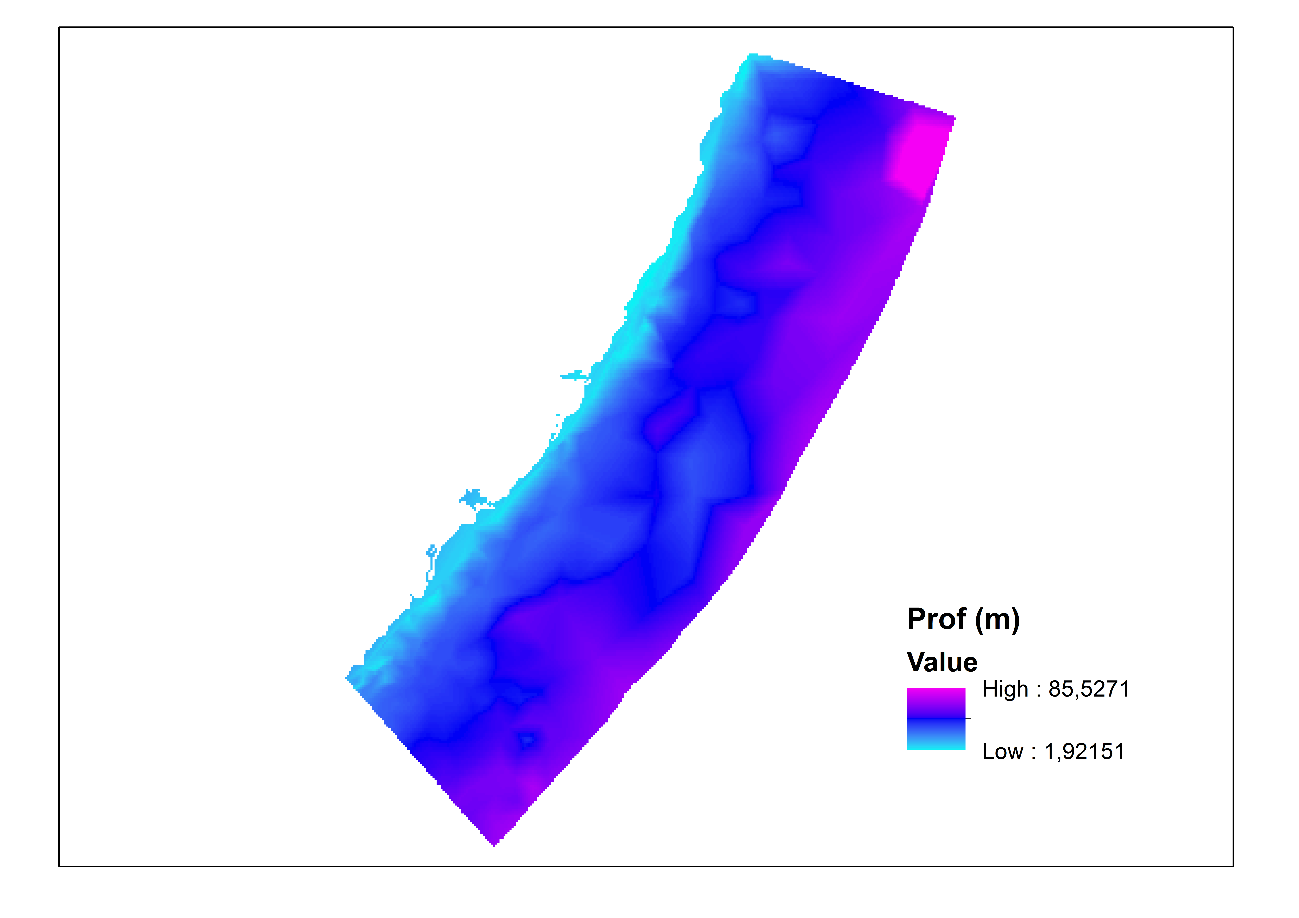


Fig S1 - Bathymetry of MPA Costa dos Corais layer used in the present study. Figure elaborated by the authors using Arcgis PRO.


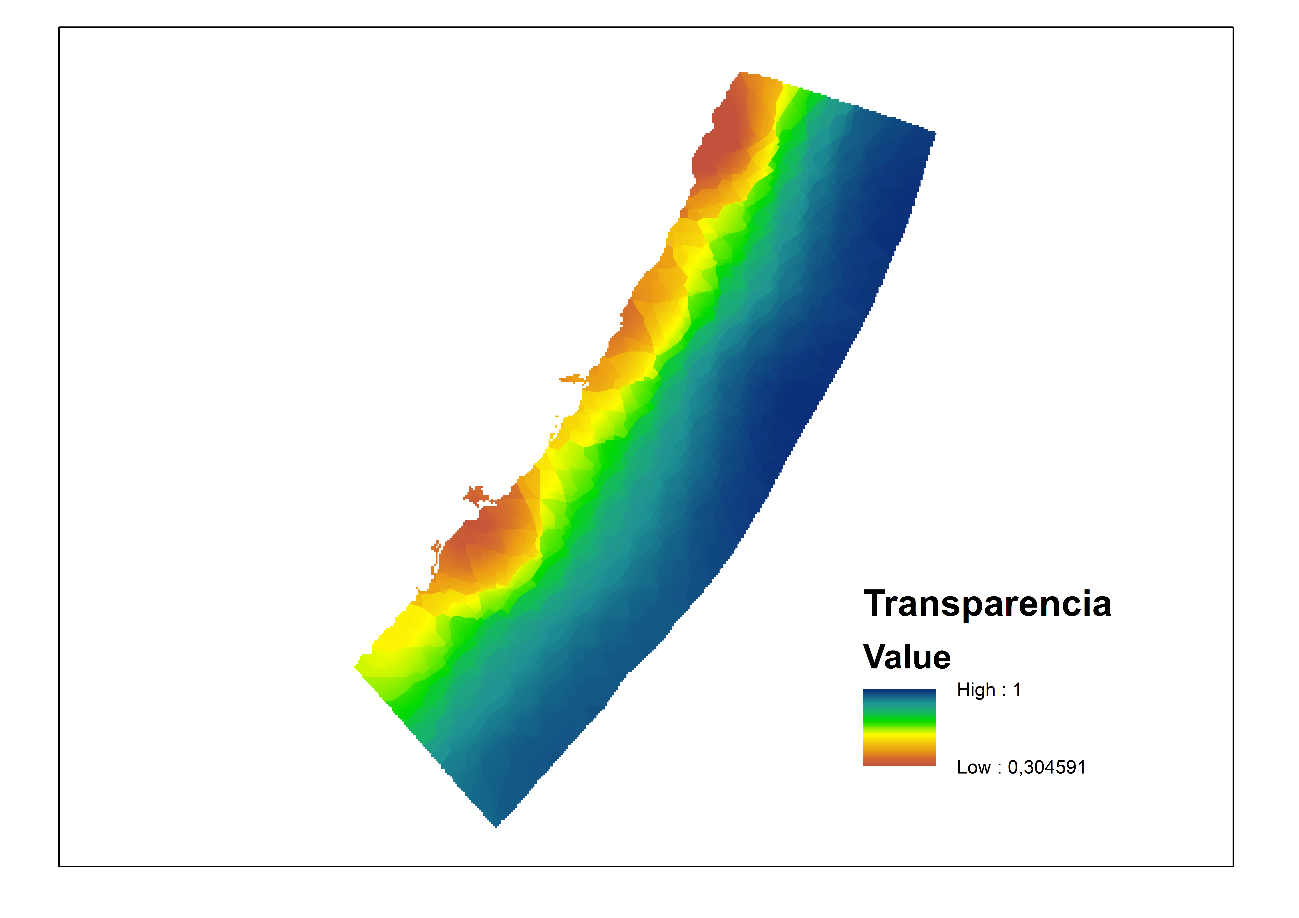


Fig S2 – Transparency of MPA Costa dos Corais layer used in the present study. Figure elaborated by the authors using Arcgis PRO.


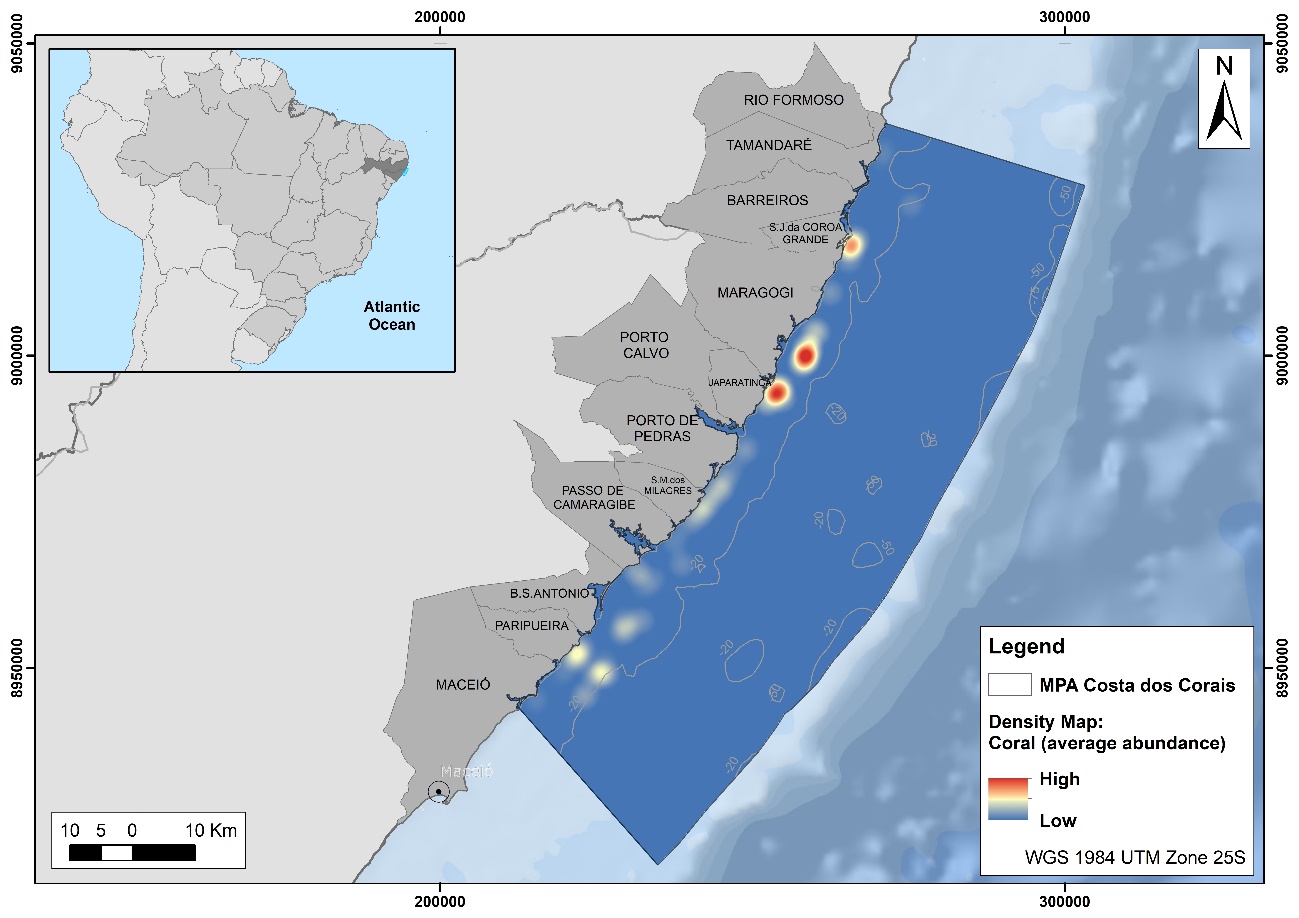


Fig S3 – Coral cover of MPA Costa dos Corais layer used in the present study. Figure elaborated by the authors using Arcgis PRO.


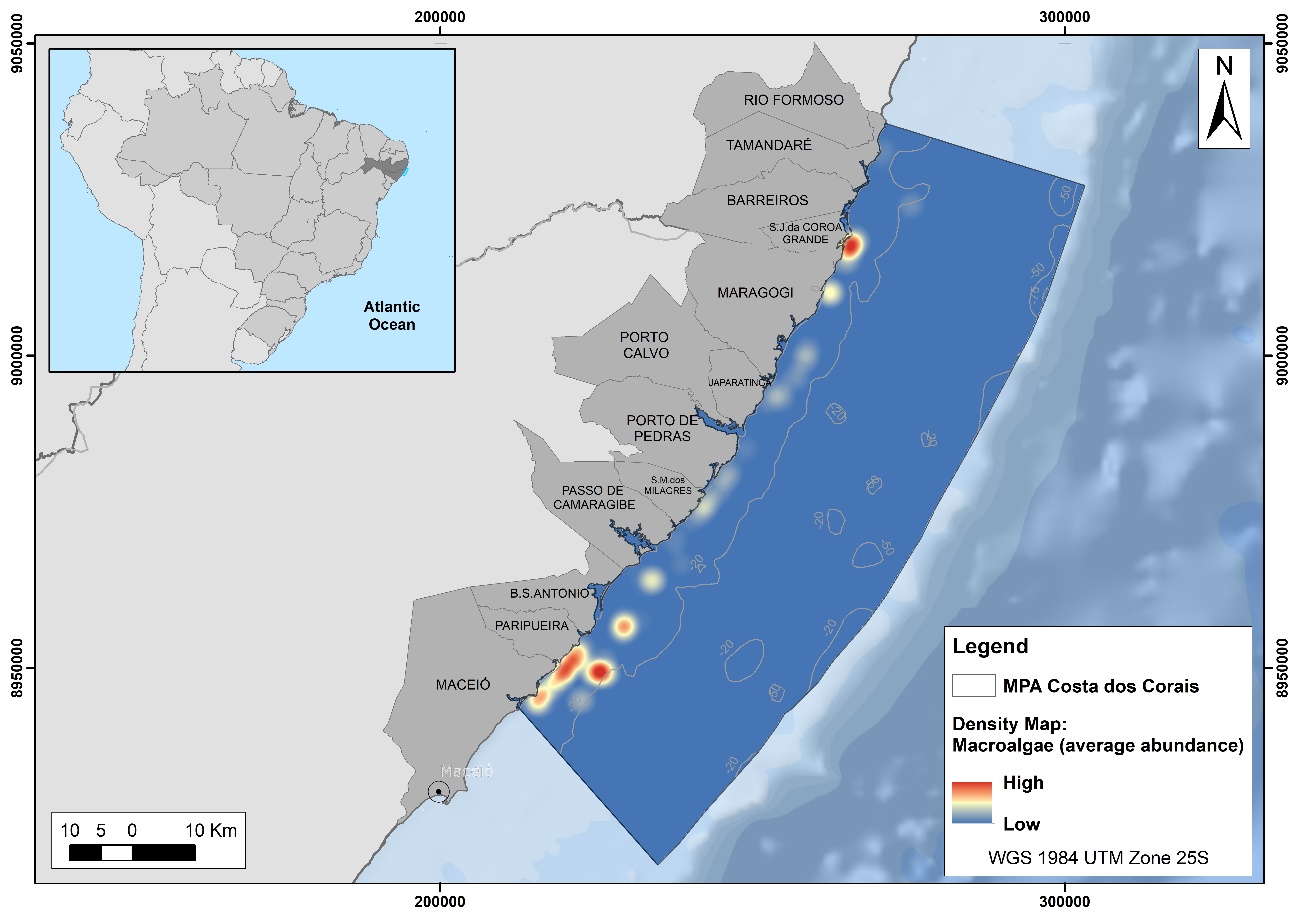


Fig S4 – Algae cover of MPA Costa dos Corais layer used in the present study. Figure elaborated by the authors using Arcgis PRO.

**Supplementary material**

**Table S1 -** Environmental variables used of parrotfish species distribution modelling (SDM) at the MPA Costa dos Corais.

| **Variable** | **Source** | **Format** | **Resolution (cell size)** |
| --- | --- | --- | --- |
| Bathymetry | ICMBio | Tiff | 99,37 x 99x37 |
| Transparency | ICMBio | Tiff | 99,37 x 99x37 |
| Coral cover | ICMBio | Tiff | 99,37 x 99x37 |
| Macroalgae cover | ICMBio | Tiff | 99,37 x 99x37 |
